# Study of Thermalization Mechanisms of Hot Carriers in BABr-Added MAPbBr_3_ for the Top Layer of Four-Junction Solar Cells

**DOI:** 10.3390/nano14242041

**Published:** 2024-12-19

**Authors:** Yi Zhang, Huilong Chen, Junfeng Qu, Jiayu Zhang, Gavin Conibeer

**Affiliations:** 1 College of Renewable Energy, Hohai University, Changzhou 213200, China; 231606050002@hhu.edu.cn (H.C.); qujf@hhu.edu.cn (J.Q.); 2 Advanced Photonics Center, Southeast University, Nanjing 210096, China; jyzhang@seu.edu.cn; 3 School of Photovoltaic and Renewable Energy Engineering, University of New South Wales, Kensington, NSW 2052, Australia

**Keywords:** hot carrier solar cells, thermalization coefficient, perovskite

## Abstract

The hot carrier multi-junction solar cell (HCMJC) is an advanced-concept solar cell with a theoretical efficiency greater than 65%. It combines the advantages of hot carrier solar cells and multi-junction solar cells with higher power conversion efficiency (PCE). The thermalization coefficient (*Q_th_*) has been shown to slow down by an order of magnitude in low-dimensional structures, which will significantly improve PCE. However, there have been no studies calculating the *Q_th_* of MAPbBr_3_ quantum dots so far. In this work, the *Q_th_* values of MAPbBr_3_ quantum dots and after BABr addition were calculated based on power-dependent steady-state photoluminescence (PD-SSPL). Their peak positions in PD-SSPL increased from 2.37 to 2.71 eV after adding BABr. The fitting shows that, after adding BABr, the *Q_th_* decreased from 2.64 ± 0.29 mW·K^−1^·cm^−2^ to 2.36 ± 0.25 mW·K^−1^·cm^−2^, indicating a lower relaxation rate. This is because BABr passivates surface defects, slowing down the carrier thermalization process. This work lays the foundation for the theoretical framework combining perovskite materials, which suggests that the appropriate passivation of BABr has the potential to further reduce *Q_th_* and make MAPbBr_3_ QDs with BABr modified more suitable as the top absorption layer of HCMJCs.

## 1. Introduction

In 1961, utilizing the detailed balance principle, Shockley and Queisser calculated that the peak achievable power conversion efficiency (PCE) for single-junction solar cells is approximately 33% [1]. To surpass the Shockley–Queisser limit, various strategies have been employed, including the use of multiple exciton generation [2], hot carrier solar cells (HCSCs) [3], intermediate band solar cells [4], and multi-junction solar cells (MJSCs) [5]. MJSCs can extend the range of photon absorption compared to single-junction solar cells, and only tandem structures have so far reached more than the S–Q limit [6]. HCSCs reduce or even prevent thermalization loss by extracting the energy of the carriers before they cool down. The integration of hot carrier collection into MJSCs holds the promise of further surpassing the efficiency limits [7]. At present, one of the main challenges for hot carrier multi-junction solar cells (HCMJSCs) is the unclear thermalization mechanisms, especially in wide-bandgap top-layer absorber materials.

The introduction of the thermalization coefficient (*Q_th_*) makes it possible to quantify the effect of the slow hot carrier thermalization process under steady-state conditions [8,9]. Ideally, the *Q_th_* value should be low and the power loss of the thermalization in the HCSCs should be reduced. Metal halide perovskites with the chemical formula of ABX_3_ (A = monovalent cations, such as Cs^+^, MA^+^, FA^+^; B = divalent cations, such as Pb^2+^; and X = halogen anions, such as I^−^, Br^−^) have a good theoretical basis in hot carrier thermalization. *Q_th_* = 0.30 ± 0.01 W·K^−1^·cm^−2^ was observed in FAPbI_3_ thin films, which was further reduced to *Q_th_* = 0.26 ± 0.02 W·K^−1^·cm^−2^ at 160 K, and the *Q_th_* values of CsMAFAPb(Br_x_I_1−x_)_3_ and MAPbI_3_ were 0.42 ± 0.02 and 0.66 ± 0.05 W·K^−1^·cm^−2^, respectively [10].

In MJSCs, metal halide perovskites have emerged as promising absorber materials, drawing interest for their straightforward fabrication techniques, extended carrier diffusion lengths, and adjustable bandgap properties. The architecture of double-junction perovskite solar cells typically involves a higher-bandgap perovskite for the top layer and a lower-bandgap layer at the bottom [11]. In solar cells with three junctions and above, the bandgap of the top absorber layer can become larger. According to the National Renewable Energy Laboratory’s cell efficiency chart, at present, the solar cells with the highest PCE (47.6%) use four-junction solar cells. In HCSCs, the phonon bottleneck effect (PBE, or hot phonon effect) has been demonstrated to be one of the most important mechanisms that could effectively slow down the thermalization process [12,13]. In this process, the hot carriers are only able to scatter with a limited number of phonon modes, which significantly prolongs the carrier lifetime. Therefore, the key to studying the mechanisms of hot carrier relaxation lies in exploring the specific mechanism of PBE [14]. To study PBE, it is necessary to first determine the degree of slowing of hot carrier cooling; that is, a decrease in the carrier relaxation rate was observed in the fitting according to the power-dependent steady-state photoluminescence (PD-SSPL) experiment.

In this work, it is theoretically proposed to combine a hot carrier solar cell with the top layer of a four-junction solar cell. The aim is to increase its potential efficiency and study the thermalization mechanism of the top layer. The thermalization mechanisms of wide-bandgap perovskites for the top-layer absorber of four-junction HCSCs are studied based on *Q_th_*. By using PD-SSPL measurements, the aim is to calculate the *Q_th_* under the two preparation methods and to study the carrier thermalization mechanism and phonon bottleneck effect in MAPbBr_3_ quantum dots. The solar cell performance has been further improved by adding butylammonium bromide (BABr), but the relaxation mechanism of MAPbBr_3_ QDs with BABr modified as the top layer of the HCMJSCs and their *Q_th_* in HCSCs have never been studied before. This study not only contributes to the understanding of the thermalization mechanisms in wide-bandgap perovskites, which is crucial for the development of high-efficiency top-layer absorbers, but also provides insights into the carrier thermalization and phonon bottleneck effects in MAPbBr_3_ quantum dots with the addition of BABr.

## 2. Experiments

The properties of MAPbBr_3_ under quantum dot structure need to be studied. This is achieved by manufacturing samples using specific method and then characterizing using various techniques such as X-ray diffraction (XRD), absorption spectrum (Abs), steady-state photoluminescence (PL), and power-dependent steady-state photoluminescence (PD-SSPL).

### 2.1. Sample Fabrication

Methylammonium bromide (MABr) and butylammonium bromide (BABr) were purchased from Xi’an Polymer Light Technology Corp. Xi’an, Shaanxi province, China. Lead bromide (PbBr_2_, 99%) was purchased from Shanghai Aladdin Biochemical Technology Co., Ltd. Shanghai, China. N,N-dimethylformamide (DMF, 99.8%) was purchased from Sinopharm Chemical Reagent Co., Ltd. Shanghai, China. Polymethyl Methacrylate (PMMA) was purchased from Shanghai Aichun Biotechnology Co., Ltd. Shanghai, China.

Method 1: Dissolve 0.025 mmol of MABr and the same mole of PbBr_2_ in 10 mL of DMF; add 1 g of PMMA. Stir at 60 °C until completely dissolved.

Method 2: Dissolve 0.025 mmol of MABr and the same mole of PbBr_2_ in 10 mL of DMF; add 0.02 mmol of BABr and 1 g of PMMA. Stir at 60 °C until completely dissolved.

The perovskite precursor was applied to the glass substrate using a blade coating method [15]. Subsequently, the glass was transferred to a low-pressure vacuum oven to evaporate the DMF at 40 °C. The sample was taken out of the vacuum oven while the film turned yellow–green.

The main difference between the two methods is whether to use the BABr or not. BABr is added to passivate surface defects, improve fluorescence intensity and lifetime, inhibit ion migration, and play a vital role in stability [16]. BA^+^ is also an ideal passivation choice due to its chemical inertness, hydrophobicity, and other properties [17].

### 2.2. Characterizations

XRD was utilized to analyze the structural characteristics of MAPbBr_3_ using a thermos scientific escalab 250xi spectrometer (Thermo Fisher Scientific, MA, U.S.) with Cu Kα as the X-ray source. The scanning speed of XRD is 5°/min and ranges from 3° to 50°. Absorption and PL spectra of samples were measured with a UV-3600 UV–vis spectrometer (Shimadzu Corporation, Kyoto, Japan) and an RF-5301PC (Shimadzu Corporation, Kyoto, Japan). To explore the carrier relaxation dynamics within solar cells, the samples were probed using PD-SSPL. The exciton is photoexcited using a pulsed laser with an excitation source of 400 nm, and the exciton emits a photoluminescence spectrum by a radiative recombination. By increasing the excitation intensity, a hot carrier population (or out-of-equilibrium carrier population) is generated, which could be detected by a change in the shape of the PL spectrum (i.e., a more obvious asymmetry can be observed at high energies). The laser spot size on the sample was measured to be 1.245 mm in diameter using a micrometer calibration grating. The PL signals were collected and measured using a Spectra Pro-300i (Acton Research Company, MA, USA) optical multi-channel analyzer.

## 3. Results and Discussion

### 3.1. XRD

**Figure 1:**
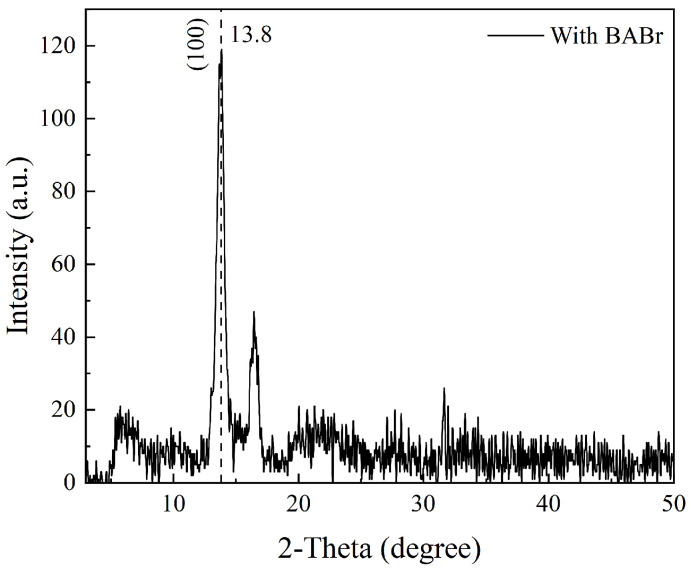
XRD pattern of MAPbBr_3_ with BABr modified.

Figure 1 shows the XRD pattern of the BABr modified- MAPbBr_3_ QDs sample, with a characteristic peak at 13.8° corresponding to (100), which is smaller relative to the 14.7° of MAPbBr_3_ derived from the other study [16]. This is due to the expansion of the lattice caused by the addition of the large-size ion BA^+^, which leads to an increase in the crystal plane spacing and a decrease in θ according to Bragg’s law. Inside the perovskite structure, the larger-cation BA^+^ than MA^+^ can form stronger non-covalent interactions with the Pb/X (X = halogen) backbone through hydrogen bonding [18], which will affect the carrier relaxation process. For more details, the hot carrier cooling is influenced by the extent of the movement of the X and Pb atoms within the [PbX6]^4−^ structure due to the interaction between the carriers and phonons [19]. The different sizes of the A-site cations can alter the lattice dimensions of A-PbBr_3_, thereby impacting their carrier thermalization process. BA^+^ has a larger radius than MA^+^; it may strongly disturb the [PbBr_6_]^4−^ framework and lead to a stronger interaction between them, leading to accelerated relaxation of HCs [18,19].

### 3.2. PL and Abs

**Figure 2:**
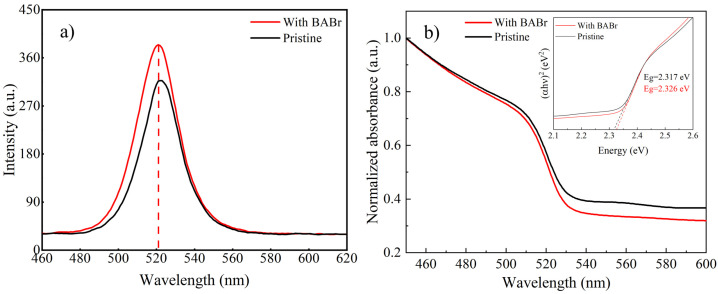
(**a**) The PL spectra for both the pristine and BABr-modified MAPbBr_3_ samples. (**b**) The UV–vis absorption spectra curves and the Tauc plot as inset in (**b**) for both samples.

The PL results shown in Figure 2 indicate that the peak position of the MAPbBr_3_ QDs at 523 nm is basically the same at 521 nm after the addition of BABr, but the fluorescence intensity after the addition of BABr is increased. The Tauc plot derived from the UV–vis absorption spectrum is utilized to illustrate the bandgap of the samples. Figure 2b indicates that the addition of BABr does not significantly alter the bandgap, which agrees with the PL results. This may be due to the coordination bonding between the amino group in the BABr molecule and the Pb^2+^ ion in MAPbBr_3_ [16], forming a stable complex that effectively passivates the defect states on the surface of MAPbBr_3_, reduces non-radiative recombination, and enables more photon-generated carriers to undergo radiative recombination, thereby increasing photoluminescence intensity.

### 3.3. SSPL

**Figure 3:**
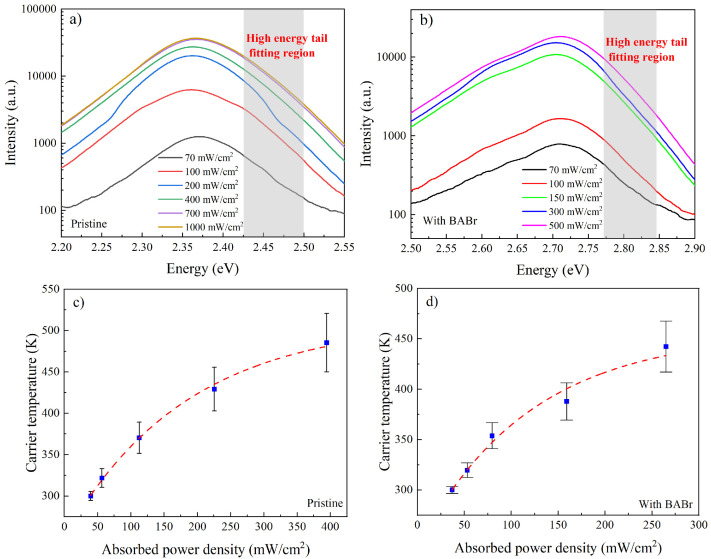
PD-SSPL results in MAPbBr_3_ QDs: (**a**) pristine and (**b**) with BABr with different power densities in mW·cm^−2^, where the high-energy-tail fitting region is indicated by the shaded area. Absorbed power-dependent carrier temperature for MAPbBr_3_ QDs (**c**) pristine and (**d**) with BABr modified calculated by high-energy-tail fitting.

The PD-SSPL results of the QDs samples prepared by two different methods are shown in Figure 3 a,b with different excitation power densities. The PL emission peaks for the pristine MAPbBr_3_ and with BABr modified can be seen at around 2.37 eV and 2.71 eV, respectively, which were higher than the bandgaps of the other dimensions [16,20]. The observed peak position deviation and the weak peak at 2.60 eV are due to the laser-induced formation of perovskite, incorporating BA into the perovskite lattice [21]. The larger size of BA compared to MA is responsible for the blue shift. If a carrier distribution similar to the Maxwell–Boltzmann model is achieved through PD-SSPL, the intensity in the high-energy tail segment adheres to an exponential decay in relation to the incident photon energy [22,23],

(1) $$I_{PL}\left(E\right)\propto \varepsilon \left(E\right)\exp\left(-\frac{E}{k_{B}T_{C}}\right)$$

where *E* is the photon energy, *ε*(*E*) is energy-dependent emissivity, and *k_B_* is the Boltzmann constant. The high-energy tail in PD-SSPL indicates the presence of carriers in high-energy states, with *T_C_* signifying the temperature distribution for these hot carriers. Moreover, in the energy regime above the emission peak, the spectra are well fitted by an exponential dependence, for which the slope on a logarithmic scale is related to the carrier temperature from Equation (1). Figure 3c,d depict the determined carrier temperatures in relation to the absorbed power density. It is observed that the carrier temperature increases with a higher absorbed power density, as denoted by the dashed blue curve. The rise in carrier temperature is likely attributed to the delayed thermal dissipation of energetic carriers at high absorbed power density.

In polar semiconductors, the primary process for carrier thermalization involves longitudinal optical (LO) phonon scattering. During this process, the energy of the carriers is transferred to LO phonons, which subsequently decay into acoustic phonons, primarily through the Klemens mechanism, with the Ridley process being a secondary route. The acoustic phonons then release their energy as lattice heat. The rate at which thermal energy is thermalized (*P_th_*) is influenced by the temperature discrepancy between the carriers and the lattice (Δ*T* = *T_C_* − 300 K), the energy of the LO phonons (*E_LO_*), and *Q_th_*, as described by Equation (2) [22,23],

(2) $$P_{th}=Q_{th}∆T\exp\left(-\frac{E_{LO}}{k_{B}T_{C}}\right)$$

where *E_LO_* for MAPbBr_3_ QDs is 24.9 meV [24]. The *Q_th_* can be calculated based on Equation (2), which is applicable when only *LO* phonon relaxation dominates, which occurs in halide perovskites and this work [25]. Due to the low rate of radiant energy of the sample, *P_th_* is approximately equal to the absorbed power density *P_abs_* (i.e., *P_abs_ = P_th_*).

**Figure 4:**
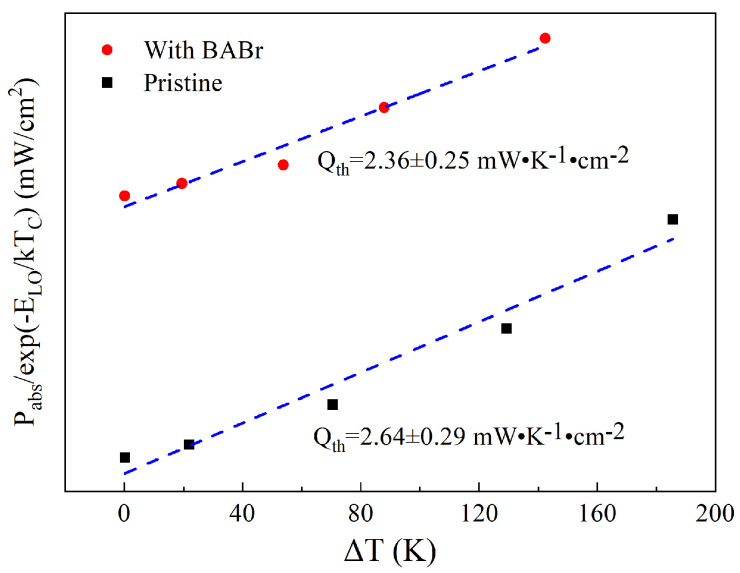
*P_abs_*/*exp*(*−E_LO_*/*k_B_T_C_*) (mW·cm^−2^) as a function of Δ*T* (K); the gradient indicated by the blue dashed line yields the thermalization coefficient *Q_th_*, with values of 2.64 ± 0.29 mW·K^−1^·cm^−2^ and 2.36 ± 0.25 mW·K^−1^·cm^−2^ for pristine and with BABr modified.

Figure 4 shows a clear linear relationship between *P_abs_*/*exp*(*−E_LO_/k_B_T_C_*) and Δ*T*, with the slope indicating the *Q_th_* value of 2.64 ± 0.29 mW·K^−1^·cm^−2^ for MAPbBr_3_ QDs, and the *Q_th_* value after passivation with BABr is relatively small, 2.36 ± 0.25 mW·K^−1^·cm^−2^. This implies that MAPbBr_3_ with BABr modified has a lower energy relaxation rate. By increasing the carrier density, the cooling rate of hot carriers can be reduced by more than an order of magnitude by utilizing the large polaron screening effect or PBE [26,27,28]. At high carrier concentrations (above 10^19^ cm^−3^), the interaction between hot phonon and auger recombination (AR) heating further delays the cooling of HC [25,29]. In PD-SSPL, the increase in incident power density improves the reabsorption of LO phonons and affects the photon dissipation process in PBE by increasing the carrier density, thereby prolonging the cooling time of the carrier temperature. Specifically, after photoexcitation, the hot carriers release excess energy into the LO phonon through non-adiabatic coupling and further dissipate into the environment. At the same time, many LO phonons are heated to a non-equilibrium state at high carrier densities induced by high power density, resulting in possible reabsorption of LO phonons [24]. As a result, hot carrier cooling slows down significantly due to PBE, making the *Q_th_* of MAPbBr_3_ QDs much smaller than that of the bulk perovskite [10]. The surface defect that can provide additional phonon scattering centers is passivated with the addition of BABr [16]. The reduced scattering centers can prolong the phonon lifetime, leading to more opportunities for phonon reabsorption, therefore further significantly reducing the thermalization rate. According to Ref. [17], BABr can also enhance the PL intensity and effectively inhibit non-radiative recombination, and the speed of the carrier recombination will be reduced, which means that the carrier lifetime will be extended. These result in a decrease in *Q_th_* after adding BABr. As mentioned above, BA^+^, with a larger cation radius than MA^+^, increases the carrier relaxation rate, which is one of the reasons why the addition of BABr does not make the *Q_th_* of MAPbBr_3_ QDs very small.

**Figure 5:**
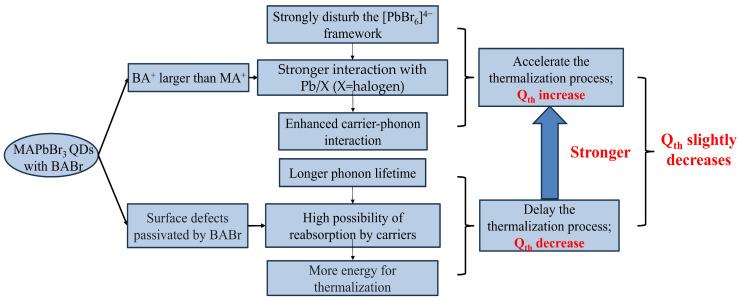
The effects of BABr addition on thermalization and *Q_th_* in MAPbBr_3_ QDs are analyzed from different perspectives.

Based on the above, the addition of BABr has different effects on the rate of retarded relaxation from different perspectives. Figure 5 shows the flow chart of its influence on the thermalization mechanism and *Q_th_*. For more details, according to the results of PD-SSPL fitting, there is still a slight decrease in *Q_th_* after the addition of BABr. Therefore, the addition of BABr in this work still plays a positive role in decreasing the carrier relaxation rate, so the goal of subsequent research is to achieve the shortest thermalization process by adjusting the addition amount of BABr and increasing the excitation power density so that PBE and AR heating become dominant [25].

## 4. Conclusions

In conclusion, the ultrafast dynamics of hot carriers in MAPbBr_3_ QDs perovskites were systematically studied, where two thermalization coefficient *Q_th_* values regarding different methods were introduced to quantitatively evaluate the relaxation rate based on the PD-SSPL. The introduction of BABr resulted in enhanced fluorescence intensity, longer carrier lifetime, and passivation of surface defects. This affects the thermalization process of hot carriers. For more details, the *Q_th_* value of the MAPbBr_3_ QDs modified by BABr was 2.36 ± 0.25 mW·K^−1^·cm^−2^ smaller than that of the original sample of 2.64 ± 0.29 mW·K^−1^·cm^−2^. It is believed that PBE and AR heating significantly slow down the thermalization process of the carriers, so the *Q_th_* in QDs should be much smaller than that in bulk perovskite, although the larger cation of BA^+^ than MA^+^ may strongly disturb the [PbBr_6_]^4−^ framework and lead to a stronger interaction between them, increasing *Q_th_*. It can also passivate surface defects and prolong the carrier and phonon lifetimes, leading to a decrease in the *Q_th_*. It is concluded that BABr can slow down carrier thermalization progress; however, a larger-cation BA^+^ delays that, so the *Q_th_* after adding BABr does not decrease too much. On the one hand, quantum dots have a small *Q_th_* value relative to bulk materials and are more suitable for acting as a top absorber layer for HCMJSCs because they have a longer time to trap hot carriers that are not relaxed to the band edge. On the other hand, the PCE values approach a maximum when *Q_th_* tends to 0, which corresponds to the absence of any thermalization loss. Conversely, the PCE value gradually approaches the S–Q limit value of PCE with an increase in *Q_th_*. Therefore, future work should adjust the amount of BABr added and the excitation power density to maximize the effect of PBE and AR in slowing down the carrier thermalization process and achieve a lower *Q_th_*, which will further significantly improve PCE in HCMJSCs. Additionally, the design and discussion regarding the overall device of the cell using such material will be carried out in subsequent studies.

## Data Availability

The data that support the findings of this study are available from the corresponding author upon reasonable request.
